# Improving Membrane Activity and Cargo Delivery Efficacy of a Cell-Penetrating Peptide by Loading with Carboranes

**DOI:** 10.3390/pharmaceutics13122075

**Published:** 2021-12-03

**Authors:** Tamara Lützenburg, Nele Burdina, Matthias S. Scholz, Ines Neundorf

**Affiliations:** 1 Institute for Biochemistry, Department of Chemistry, University of Cologne, Zülpicher Str. 47a, 50674 Cologne, Germany; tluetze3@uni-koeln.de (T.L.); nburdina@smail.uni-koeln.de (N.B.); 2 Pharmaceutical Chemistry I & II, Pharmaceutical Institute, Faculty of Mathematics and Natural Sciences, University of Bonn, An der Immenburg 4, 53121 Bonn, Germany; scholz@uni-bonn.de

**Keywords:** alpha-helix stabilization, secondary structure, cell-penetrating peptides, lipid–peptide interaction, peptide engineering

## Abstract

Cell-penetrating peptides (CPPs) have emerged as versatile tools to increase the intracellular accumulation of different kinds of cargoes. For an efficient cellular uptake and drug delivery, their organization into a distinct and stable secondary structure at the outer surface of the plasma membrane is a hallmark and supports optimal lipid–peptide interactions. Incorporation of hydrophobic moieties, such as carboranes (CBs), has the potential to increase the lipophilicity of peptides, and thus, to facilitate the formation of secondary structures. Herein, we present synthesis and biophysical as well as biological characterization of carborane-CPP conjugates having incorporated one or more CB clusters. Our results highlight the possibility to modulate the secondary structure of CPPs by the addition of CB’s leading to constructs with altered membrane activity and promising use in terms of nucleic acid delivery.

## 1. Introduction

Biologically active peptides often develop distinct and stable conformations when in proximity to their binding partners. In case of cell-penetrating peptides (CPPs), structural organization is essential for efficient interaction with components of the plasma membrane [1]. This structuring readily occurs when CPPs are in close proximity to the lipid bilayer [2,3] and induces the following internalization process [4,5]. Consequently, chemical modifications that evoke and stabilize such secondary structures are of paramount importance to enhance the intracellular accumulation of CPPs.

During the past years, CPPs have emerged as promising delivery systems for small drug molecules, nanoparticles, nucleic acids or proteins [6,7,8,9,10,11], and have been widely applied for, e.g., anticancer treatments [12]. The formation and stability of their secondary structure is influenced by numerous factors including sequence length and amino acid composition [13,14]. For instance, strategies to increase the internalization efficiency of CPPs include the introduction of modifications or substitution of distinct amino acids within the peptide sequence [15,16]. Interestingly, it has been found out that the presence of hydrophobic amino acids, or the attachment of other hydrophobic moieties, e.g., lipidation, might further induce superior interaction with lipid bilayers [15,17,18]. In this respect, so-called car(ba)boranes (CBs) have emerged as interesting lipophilic substitutes and were already integrated as pharmacophores into different small molecule drugs. In addition, the incorporation of carboranes into therapeutic peptides was already described [19,20,21]. In this regard, CB-peptide conjugates turned out to be promising for application in tumor-selective boron neutron capture therapy (BNCT), and also CPPs were used to create such boron delivery systems [22,23]. However, to our knowledge, it is not clear and has not been elucidated yet how carboranes influence the physicochemical and biological characteristics of a CPP, and if one can use CBs to modulate the potential and activity of CPPs. Recently, we reported the straight-forward synthesis of peptide conjugates containing one CB cluster [24]. In this present study, we extended our efforts to engineer and modulate CPPs having one or more boron clusters incorporated. As a model CPP, we used sC18, which was developed in our group and turned out as a useful transporter for many different cargoes [25,26,27,28,29]. We studied the physicochemical properties of the new CB_x_-CPP conjugates, as well as their biological activity when in contact with different membrane models. In a preliminary proof-of-principle study, we confirmed the cargo delivery potential of CB_4_-sC18, which proved to be highly promising as a transporter for pDNA.

## 2. Materials and Methods

### 2.1. Carborane Synthesis

The carborane building block (1,7-dicarba-closo-dodecarbaborane) was purchased from Katchem Ltd. (Prague, Czech Republic). Additional chemicals used were bought from Acros (Geel, Belgium), Alfa Aesar (Haverhill, MA, USA), Fluka (Taufkirchen, Germany), Merck (Darmstadt, Germany), Sigma-Aldrich (Merck group: Darmstadt, Germany), and Carbolution (St. Ingbert, Germany). *Meta*-carborane-1-carboxylic acid was synthesized according to published procedures [24].

### 2.2. Peptide Synthesis

All used amino acids were purchased from IRIS Biotech (Marktredwitz, Germany) and were bought as Nα-Fmoc-protected versions. Additional chemicals and consumables required for synthesis were derived from Fluka (Taufkirchen, Germany), Merck (Darmstadt, Germany), Sarstedt (Nümbrecht, Germany), Sigma-Aldrich (Merck group: Darmstadt, Germany), and VWR (Darmstadt, Germany). This includes ethyl cyano(hydroxyimino)acetate (Oxyma), *N*,*N*′-diisopropylcarbodiimide (DIC), 1-[bis(dimethylamino)methylene]-1H-1,2,3-triazolo[4,5-b]pyridinium 3-oxid hexafluorophosphate (HATU), *N*,*N*-diisopropylethylamine (DIPEA), acetonitrile (ACN), and trifluoroacetic acid (TFA). For peptide synthesis, a combination of standard Fmoc/*t*-Bu solid-phase peptide synthesis (SPPS) on a Syro I peptide synthesizer (MultiSynTech, Bochum, Germany) and manual coupling protocols were used [25]. Peptides were generated as *C*-terminally amidated molecules on a Rink amide resin. The identity of peptides was confirmed via HPLC-ESI mass spectrometry (LTQ XL, Thermo Scientific, Waltham, MA, USA).

### 2.3. Carboranyl-Peptide Synthesis

Synthesis of carborane-peptide conjugates was carried out on solid support (loading 0.5 mmol/gram resin). The carborane cluster was introduced using 2 eq activated *meta*-carborane-1-carboxylic acid with 5 eq Oxyma/DIC overnight at room temperature. Carboranyl-peptides were cleaved from the solid support using trifluoroacetic acid/triisopropylsilane/water (95/2.5/2.5, *v*/*v*/*v*). Identification was performed via HPLC-ESI (Chromolith^®^ Performance RP-18e, 100-4.6 mm, Merck, Darmstadt, Germany; 10–60% ACN in water (incl. 0.1% formic acid) over 15 min; 0.6 mL/min flow rate) mass spectrometry. Purification was carried out using preparative HPLC (Nucleodur C18ec; 100-5; Macherey-Nagel, Düren, Germany), with a flow rate of 1.5 mL/min over 45 min. Different gradients were used, according to the hydrophobicity of the conjugate; CB_1_-sC18: 10–60% ACN in water; CB_2_-sC18: 20–70% ACN in water; CB_3_-sC18, CB_4_-sC18, CB_5_-sC18: 30–90% ACN in water. Water was supplemented with 0.1% TFA, ACN with 0.08% TFA. Final chromatograms were recorded using a linear gradient from 20–80% ACN in water (incl. 0.1% TFA) using a Nucleodur C18ec; 100-5 (Macherey-Nagel, Düren, Germany) column (1 mL/min flow rate).

### 2.4. Circular Dichroism Spectroscopy

Conjugates were dissolved in 10 mM phosphate buffer (pH 7.0), either alone or supplemented with 50% TFE (*v*/*v*), to a final concentration of 20 µM. CD spectra of all conjugates were recorded from 180–260 nm (0.5 nm intervals) on a J-715 spectropolarimeter (JASCO, Pfungstadt, Germany) in an N_2_ atmosphere.

### 2.5. Cell Viability Assay

In total, 17.000 HeLa cells per well were seeded onto 96-well plates (Sarstedt, Nümbrecht, Germany). After confluency of 80–90% was reached, the growth medium was removed, and cells were incubated with 100 µL of peptide solution of different concentrations (125 nM–100 μM in serum-free medium) for 24 h at 37 °C. For a positive control, cells were treated with 70% EtOH for 10 min. Cells were washed with serum-free medium and incubated with 10% (*v*/*v*) resazurin (Sigma-Aldrich, Merck group: Darmstadt, Germany) in serum-free medium for 1.5 h at 37 °C. Cell viability was determined by measurement of the resorufin product at 595 nm (λ_ex_ = 550 nm) on a Tecan infinite M200 plate reader (Tecan Group AG, Männedorf, Switzerland). Viability was calculated from the fluorescence values of treated cells in relation to untreated cells.

### 2.6. CF-Leakage Assay

Large unilamellar vesicles (LUVs) were prepared by dissolving different lipids in CHCl_3_, to reach the desired compositions (lipids were purchased from Avanti Polar Lipids, Inc., Alabaster, AL, USA). To remove the solvent, the mixture was placed into a round-bottomed flask on ice and the solvent was evaporated under reduced pressure for 20 min at 40 °C and afterwards under vacuum for another 5 min. The dried lipid film was then hydrated with HKS buffer (25 mM HEPES, 150 mM KCl, pH 7.4, 10% sucrose (*w*/*v*)), supplemented with 100 mM 5(6)-carboxyfluorescein (CF), and homogenized for 5 min at 45 °C to form liposomes (final concentration: 8 mM). The resulting multilamellar vesicle dispersion underwent 10 freeze/thawing cycles and was passed 21 times through a mini extruder (0.4 µm polycarbonate treck-etch membrane; Avanti Polar Lipids, Inc., Alabaster, AL, USA). External CF was removed by size exclusion chromatography (PD10 column, GE Healthcare, Chicago, IL, USA). Peptides were added to the LUVs and the CF release was monitored by measuring the increased fluorescence intensity on a plate reader (Biotek, Winooski, VT, USA). After 90 min, Triton-x-100 was added to a final concentration of 0.4% (*v*/*v*) to measure the maximal leakage and normalize data.

### 2.7. Hemolysis Assay

Human red blood cells were washed five times with PBS (4 °C, 3000× *g*, 5 min) and diluted to 5% (*v*/*v* in PBS). In total, 100 µL of this solution were transferred into a fresh 96-well plate. In total, 50 µL of peptide solution were added and gently resuspended. Red blood cells were incubated for either 1 or 24 h at 37 °C and 5% CO_2_, respectively. In total, 50 µL of 10% Triton-x-100 were added 10 min prior to the end of the incubation time and served as a positive control. After incubation, cells were centrifuged (r.t., 2500× *g*, 3 min). The supernatant was carefully transferred into a fresh 96-well plate and the absorption at 560 nm was measured on a Tecan infinite M200 plate reader (Tecan Group AG, Männedorf, Switzerland). The measured absorption referred to the hemoglobin concentration.

### 2.8. Flow Cytometry

HeLa cells were seeded onto 24-well plates (Sarstedt, Nümbrecht, Germany, 100,000 cells per well, respectively). After reaching 80–90% confluency, medium was removed, and cells were incubated with 1 µM CF-labeled peptide solution in serum-free medium for either 30 min or 2 h at 37 °C. The peptide solution was removed, cells were washed twice with PBS, and trypsinized for 5 min. Detached cells were resuspended in full medium (phenol red free) and afterwards analyzed using a guava easycyte HT flow cytometer (Merck, Darmstadt, Germany).

### 2.9. Confocal Laser Scanning Microscopy (CLSM)

HeLa cells were seeded into 8-well plates (ibidi, Gräfelfing, Germany, 40,000 cells per well, respectively) and grown to 80–90% confluency. Cells were treated with 1 µM CF-labeled peptide in serum-free medium for 30 min or 2 h at 37 °C. Ten minutes prior to the end of the incubation time, nuclei were stained with Hoechst33342. The peptide solution was removed, and cells were treated with trypan blue (150 mM in 0.1 M acetate buffer, pH 4.15) for 30 s. Cells were washed twice with PBS and covered with fresh medium supplemented with FBS. Microscopic analysis was performed using an inverse confocal TCS SP8 microscope (Leica Microsystems, Wetzlar, Germany), equipped with a 63× oil-immersion objective. Images were recorded with LAS X software (Leica Microsystems, Wetzlar, Germany) and adjusted with Fiji.

### 2.10. Critical Micelle Concentration (CMC) Determination

Peptide conjugates were dissolved in water and different concentrations (1–200 µM) were prepared. UV-VIS spectra ranging from 200–700 nm were recorded using a 1 cm path length quartz cuvette in a Spectroquant Pharo 300 photometer (Merck, Darmstadt, Germany). The absorbance at 255 nm was plotted against the logarithm of the peptide concentration. The intersection point of two lines was determined as CMC.

### 2.11. Proliferation Assay

In total, 3000 HeLa cells per well were seeded onto 96-well plates (Sarstedt, Nümbrecht, Germany). When a confluency of 30–40% was reached, medium was removed and cells were incubated with peptides and Daunorubicin at different concentrations (1 µM peptide concentration and 100 nM or 1 µM Daunorubicin in serum-free medium) for 2 h at 37 °C. Afterwards, a washout was performed, and cells were washed once with 100 µL of serum-free medium. Then, 200 µL of medium with serum were added and cells were grown for 72 h at 37 °C. As a positive control, cells were treated with 70% EtOH for 10 min. Afterwards, cells were covered with 10% (*v*/*v*) resazurin (Sigma-Aldrich, Merck group: Darmstadt, Germany) in serum-free medium for 1.5 h at 37 °C. The cell viability of treated cells was determined relative to that of untreated cells by measurement of the resorufin product at 595 nm (λ_ex_ = 550 nm) on a Tecan infinite M200 plate reader (Tecan Group AG, Männedorf, Switzerland). Viability was calculated from the fluorescence values of treated cells in relation to untreated cells.

### 2.12. Electrophoretic Mobility Shift Assay (EMSA)

Peptides were co-incubated with mCherry plasmid at various ratios for 1 h at 37 °C in nuclease-free water (total volume: 15 µL). The following mass ratios were chosen (peptide:plasmid): 0.25:1, 0.5:1, 1:1, and 3:1 (mCherry plasmid: 250 ng). After incubation, 3 µL of 6x Loading Dye were added into the reaction mixture and the mixture was loaded onto a 1% agarose gel. The agarose gel was run for 1.5 h at 100 V and the gel analyzed in a ChemiDoc XRS (Bio-Rad, Hercules, CA, USA). 

### 2.13. Transfection with mCherry Plasmid

HeLa cells were seeded into 8-well plates (ibidi, Gräfelfing, Germany, 8000 cells per well, respectively) and grown to 30–40% confluency. Peptides and mCherry plasmid were co-incubated for 1 h at 37 °C at a ratio of 3:1 in serum-free medium (mCherry plasmid: 250 ng). Afterwards, the medium was removed, and the solution was added to the cells. Additional serum-free medium was added to ensure a final peptide concentration of 1 µM. Cells were incubated for 2 h with the peptides and peptide/plasmid solution at 37 °C, respectively. As a positive control, Lipofectamine 2000 (Invitrogen, Thermo Scientific, Waltham, MA, USA) was used and as a negative control, cells were treated with plasmid only. Afterwards, a washout was performed, and cells were washed once with 150 µL of serum-free medium. In total, 300 µL of medium with serum were added and cells were grown for 72 h at 37 °C. Ten minutes prior to microscopic analysis, 0.6 µL of Hoechst33342 were added. Then, cells were washed twice with 150 µL of serum-free medium. In total, 300 µL of serum-supplemented medium were added and cells analyzed using a BZ-X800E microscope (Keyence, Osaka, Japan).

## 3. Results

### 3.1. Synthesis of Carborane-Peptide Conjugates 

**Scheme 1:**
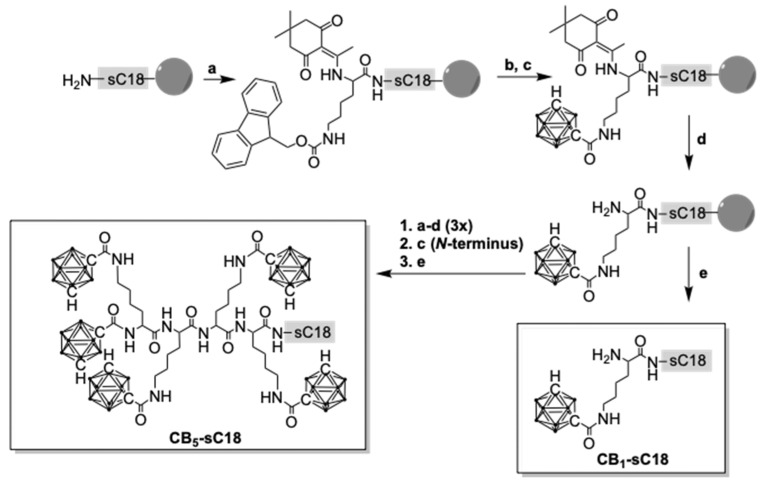
Synthesis of CB-sC18 conjugates; a: Dde-Lys(Fmoc)-OH, HATU, DIPEA; b: 30% piperidine/DMF; c: *meta*-carborane-1-carboxylic acid, Oxyma, DIC; d: 3% hydrazine/DMF; e: TFA/TIS/water (95/2.5/2.5, *v*/*v*/*v*).

First, we synthesized a series of CB-CPP conjugates bearing up to five *meta*-CBs by using a combination of automated and manual solid-phase peptide synthesis (SPPS). In order to couple CB stepwise to sC18, additional lysines, e.g., Dde-Lys(Fmoc)-OH, were introduced (Scheme 1).

Moreover, a second fluorescent series was synthesized with all conjugates labeled with 5(6)-carboxyfluorescein (CF) at the ε-amino group of lysine at position 12 of sC18. All products were obtained in high purities and confirmed via liquid chromatography mass spectrometry analysis (Table 1, Table S1 and Figures S1–S12).

### 3.2. Physicochemical Properties of CB-CPP Conjugates

**Figure 1:**
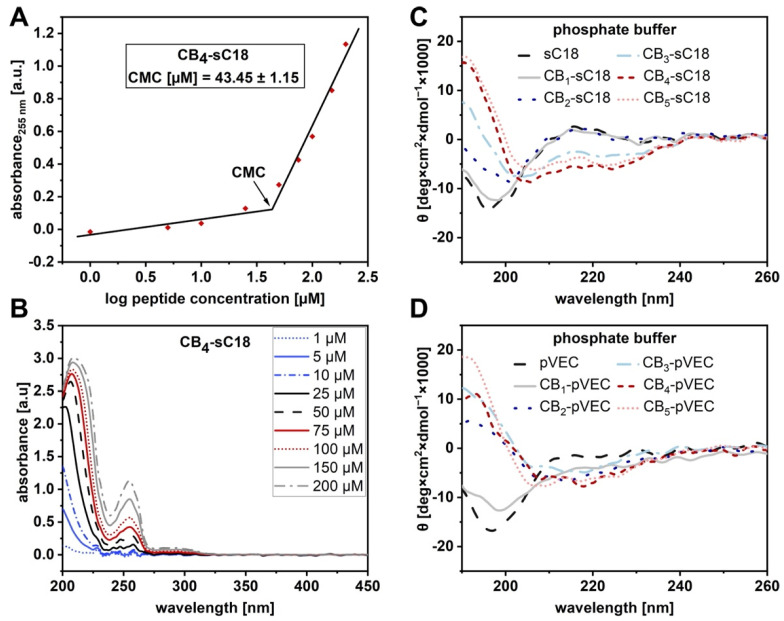
Determination of CMC and CD spectra of CB-CPP conjugates. (**A**) The determination of CMC for CB_4_-sC18 is shown by measuring the UV-VIS spectra at various concentrations (**B**). (**C**) CD spectra of CB-sC18 conjugates in phosphate buffer. (**D**) CD spectra of CB-pVEC conjugates in phosphate buffer.

Comparing the HPLC retention profiles of all CB-sC18 conjugates revealed a stepwise shift towards higher retention times dependent on the number of CBs coupled (Figure S13). Since this effect was obviously dependent on the hydrophobic nature of the conjugates, we were then interested in whether they would also self-assemble and form nanostructures, such as micelles or fibrils. An easy to apply and fast method makes use of changes in spectroscopic properties associated with micelle formation [30]. For this, one measures the UV absorbance at a specific wavelength as a function of the concentration. Because aggregates like micelles and free molecule species contribute to a different extent to the absorbed UV light, an abrupt change of the slope occurs above a certain point. The corresponding concentration is defined as the critical micelle concentration (CMC). We measured the UV-VIS spectra of all CB-sC18 conjugates and observed for all an additional concentration-dependent maximum at 255 nm (Figure 1A,B and Table 2), whereas this maximum was not detected for meta-carborane-1-carboxylic acid (Figure S14) or sC18 alone (Figure S15).

Thus, we hypothesized this maximum as evidence for a possible self-assembly, and by plotting the absorbance at 255 nm against the logarithm of the peptide concentration, we assigned the intersection point as CMC. Interestingly, determination of a CMC was only feasible for CB_3–5_-sC18 variants (Table 2, Figures S16–S19), letting us assume that self-assembly accompanied by a change in secondary structure would only be observed when three or more CBs were attached.

To find out more, we performed CD spectroscopy for all peptides solved either in phosphate buffer or in phosphate buffer supplemented with trifluoroethanol (TFE), respectively. All conjugates and sC18 adopted an alpha-helix, when present in phosphate buffer with TFE (Figure S20). On the other hand, when CB-conjugates were solved in phosphate buffer only (Figure 1C), some of them directly formed distinct secondary structures. For instance, CB_2_-sC18 and more clearly CB_3_-sC18 did not display a clear random coil structure, but the shapes of their CD curves changed to ones with a more alpha-helical character, and for CB_4_- and CB_5_-sC18, the alpha-helical structure was even more evident (Table 2). Taking this observation into account, we concluded that the number of CB moieties actually promoted the formation of alpha-helical structures. This effect might be further explained by the helical wheel projection of each conjugate showing that the hydrophobic face of the peptide was clearly enlarged when three or more CB units were attached (see Figure S21).

In order to verify the observed influence of CBs, we chose the CPP pVEC (LLIILRRRIRKQAHAHSK) [33] and synthesized the analogous series of CB_1–5_-pVEC conjugates by introducing CBs in the same way as described before (Figures S22–S27). Again, we observed that the elution profile of the different conjugates was shifted towards higher retention times, similarly as we have seen it before for the CB-sC18 series (Figure S28). More interestingly, CD spectroscopy measurements indicated a change in the secondary structure when two or more CBs were attached, while a more distinct alpha-helical structure was recognized for CB_4_- and CB_5_-pVEC (Figure 1D and Figure S29, Table 2). In conclusion, the attached CBs contribute to the formation of defined secondary structures, which is important in light of the membrane activity of CPPs.

### 3.3. Interaction with Artificial and Biological Membranes

**Figure 2:**
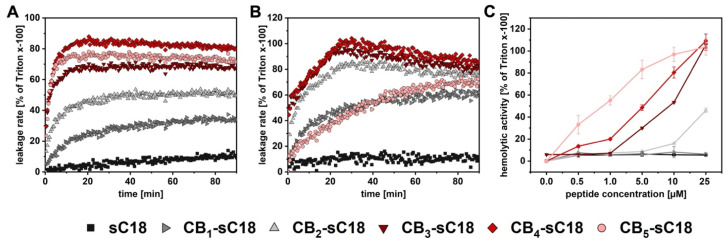
Time-dependent release of CF from negatively charged (**A**) or zwitterionic LUVs (**B**). Data were normalized to Triton x-100 control. (**C**) Hemolytic activity of CB-sC18 conjugates towards RBCs. Data were normalized to the control. Experiments were conducted in duplicate (**A**,**B**) or triplicate (**C**) with *n* = 2.

Our results thus far inspired us to investigate how the novel CB-conjugates would perform in a lipid environment. Therefore, we first used large unilamellar vesicles (LUVs) composed of different phospholipids as suitable model systems for mimicking different types of cell membranes. We prepared negatively charged (DOPC/DOPE/DOPG; 40/30/30) LUVs, representing cancer cell membranes, and zwitterionic (DOPC/DOPE; 50/50) ones, representing healthy cell membranes, and incubated them at 25 °C for 90 min with 1 µM peptide solutions (Figure 2). Both lipid systems were additionally loaded with CF in order to monitor membrane leakage via fluorescence dye outflow.

As depicted in Figure 2A, sC18 did not cause any leakage to negatively charged LUVs, while all conjugates bearing carboranes led to an increased outflow of CF, particularly CB_3_-, CB_4_-sC18, and CB_5_-sC18. The most potent conjugate was CB_4_-sC18, exhibiting not only the highest leakage rate but also the most rapid release of CF. Interestingly, this effect also appeared when CB_4_-sC18 was incubated with zwitterionic LUVs (Figure 2B), followed by CB_3_-sC18 and CB_2_-sC18. Notably, CB_5_-sC18 demonstrated the same leakage profile as CB_1_-sC18, and was, therefore, not as potent as it was towards negatively charged LUVs. This finding might be allocated to the formation of micelles and/or a higher tendency to tightly interact with the lipid phase, also leading to slight precipitation over longer time incubations. In summary, CB_4_-sC18 exhibited the highest leakage rate towards both tested model systems, while interaction with negatively charged membranes seemed to be stronger and more rapid.

Next, we incubated human red blood cells (RBCs) with different concentrations of CB-sC18 conjugates at 37 °C for 1 or 24 h, respectively. Since their cell membranes are mainly composed of neutral lipids, RBCs serve as an excellent biological model for the lytic activity of a peptide. As depicted in Figure 2C, sC18 and CB_1_-sC18 did not exhibit any effect on RBCs after 24 h of incubation even at the highest concentration used (25 µM). For sC18, this agreed with the detected low activity when in contact with zwitterionic LUVs. Compared to this, CB_2_-sC18 and all other conjugates were able to lyse RBCs, exhibiting up to 100% hemolytic activity. Interestingly, the efficacy of the conjugates to lyse RBCs increased with the number of CB attached (CB_2_-sC18 < CB_3_-sC18 < CB_4_-sC18 < CB_5_-sC18). The same activity profile was observed when incubating peptide conjugates with RBCs for only one hour (Figure S30), whereas the effects started at concentrations higher than 5 µM. Altogether, we concluded all CB-sC18 conjugates had high membrane activities. However, this property is impressively dependent on the number of CBs attached to the CPP.

### 3.4. Cytotoxicity and Cellular Uptake Studies

**Figure 3:**
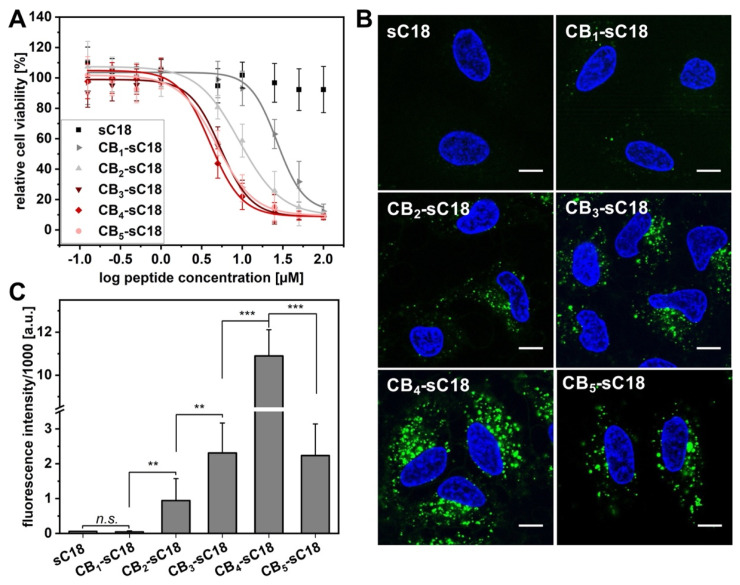
Cytotoxicity and cellular uptake into HeLa cells. (**A**) Cytotoxicity profile of CB-peptide conjugates towards HeLa cells. (**B**) CLSM analysis of 1 µM CF-labeled peptide conjugates after 2 h of incubation in HeLa cells; scale bar: 10 µm. (**C**) Flow cytometry analysis of 1 µM CF-labeled peptide conjugates after 2 h of incubation in HeLa cells. Significances were determined by Student’s *t*-test (** *p* < 0.005; *** *p* < 0.0005). CB_4_-sC18 is significant (*** *p* < 0.0005) to all conjugates.

Following, CB-sC18 conjugates’ interaction with living cells was of interest. We chose HeLa cells, since we have already used them in many studies including sC18 [25,34]. We first assessed the cytotoxicity of the novel peptides towards this cell line (Figure 3A). As expected from our previous studies, sC18 exhibited no cytotoxicity when incubated for 24 h with the cells [35]. In contrast, a clear decrease in cell viability was visible for all novel CB-sC18 conjugates (Figure 3A). While the cytotoxic activity increased from CB_1_-sC18 to CB_2_-sC18, conjugates CB_3_-, CB_4_-, and CB_5_-sC18 showed nearly the same activity profile, concluding that there is no direct correlation between the number of CBs coupled to the peptide and their cytotoxic properties. Nevertheless, the observed cytotoxic profiles agreed with the LUV experiments using negatively charged vesicles, where we measured a very fast dye release for all three conjugates CB_3_- to CB_5_-sC18, presumably due to membrane distortion. Since for both controls alone (*meta*-carborane-1-carboxylic acid as well as sC18) nearly no cytotoxicity was detected (Figure S31), we allocated this high membrane activity to the higher alpha-helical content in combination with the enlarged hydrophobic face within the CB-conjugates that was present after introducing CBs to sC18.

Next, we investigated the cellular uptake of the different CB-sC18 conjugates in HeLa cells. We performed microscopy studies using confocal laser scanning microscopy (CLSM), and quantified the uptake by flow cytometry (Figure 3B,C). Since it has been previously demonstrated that CPP uptake might be influenced by the choice of fluorophore and its position within the CPP sequence [36,37], we first assessed whether it would make any difference if sC18 was labeled with CF at the *N*-terminal or at position 12. As depicted in Figure S32, we did not determine any impact on the internalization ability of the peptide, verifying position 12 of sC18 as suitable for CF labeling. However, all conjugates were able to internalize into HeLa cells already after 30 min of incubation time and at non-toxic concentrations of 1 µM (Figure S33). The control peptide sC18 as well as CB_1_-sC18 were internalized nearly to the same extent. Again, cellular uptake increased when more CBs were attached to the peptide, whereas it was highest for CB_4_-sC18 and decreased again for the most hydrophobic conjugate CB_5_-sC18. Additionally, all conjugates showed a vesicular distribution within the cytosol of the cells, which was even more visible, when incubating the cells for 2 h (Figure 3B). This observation might be a hint of an endocytotic internalization pathway. Moreover, as illustrated in Figure 3C, CB_4_-sC18 was again internalized the most, and the cellular uptake significantly increased for all conjugates when incubating cells for 2 h, besides for CB_1_-sC18. Particularly, CB_3–5_-sC18 were internalized to a much higher extent when incubating the cells for 2 h. This might be explained by their highly increased hydrophobic character, and therefore, stronger and more intense interaction with the hydrophobic membrane core. The general decreased uptake of CB_5_-sC18 might be a result of formed self-assemblies, which are presumably taken up to a lesser extent or need more time for internalization. All in all, these results emphasized again that the attachment of CBs highly supported and adjusted the cellular uptake of all conjugates, highlighting CB_4_-sC18 as the most active candidate.

### 3.5. Cargo Delivery Studies

Lastly, we were interested in whether the CB-sC18 conjugates would be useful for intracellular cargo delivery. We chose two different cargoes, daunorubicin (Dau), which has a reasonably good cell membrane permeability by itself, as well as plasmid DNA encoding for the red fluorescent protein mCherry, in order to analyze if CB-sC18 conjugates were able to support the intracellular accumulation in HeLa cells of otherwise non-cell-permeable molecules. Since CB_4_-sC18 performed the best, but CB_1_-sC18 exhibited the lowest cytotoxic effect, we used both conjugates for our studies. Furthermore, we were interested in whether the conjugates could be applied as delivery enhancers by just co-incubating the cargo with transporter, e.g., the CB-CPP conjugate.

**Figure 4:**
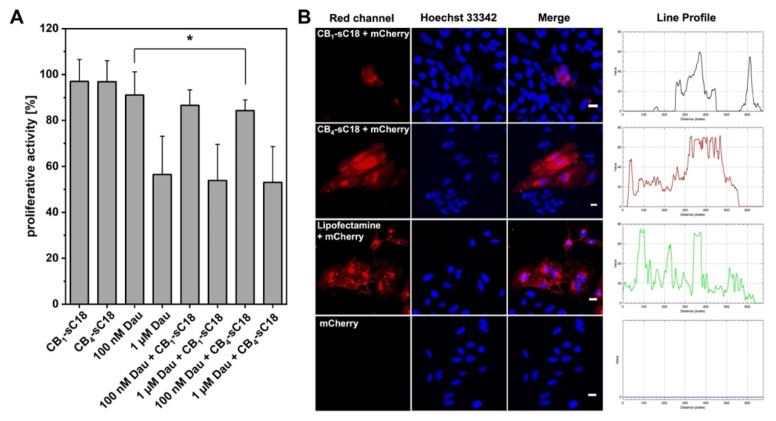
Cargo delivery properties of CB-sC18 conjugates. (**A**) Proliferative activity of CB-sC18 conjugates, Dau and CB-sC18/Dau mixture towards HeLa cells. Significances were determined by Student’s *t*-test (* *p* < 0.05). (**B**) Microscopic analysis of HeLa cells transfected with mCherry encoding pDNA using either CB_1_-sC18, CB4-sC18, or lipofectamine 2000, scale bar: 20 µm.

Our results demonstrated that both CB_1_-sC18 as well as CB_4_-sC18 did not alter the cytotoxicity of Dau. Moreover, daunorubicin’s activity was slightly increased using 100 nM Dau in combination with CB_4_-sC18 (Figure 4A). Of more interest was that CB_4_-sC18 proved to be versatile to support the internalization of pDNA encoding for mCherry. As depicted in Figure 4B, when cells were inspected 72 h after transfection, we detected no fluorescent signal for the control cells (pDNA alone) and only a weak red signal when the cells were incubated with a mixture of CB_1_-sC18 and mCherry pDNA. Instead, cells that were transfected with a solution of CB_4_-sC18 and mCherry pDNA demonstrated strong red intracellular signals derived from a successful plasmid transfection. Having a closer look at the intensity values of fluorescent cells, we observed an almost similar intensity of lipofectamine-transfected cells compared to cells that were covered with CB_4_-sC18 and mCherry pDNA (see the line profiles in Figure 4B and Figure S35).

## 4. Discussion

**Figure 5:**
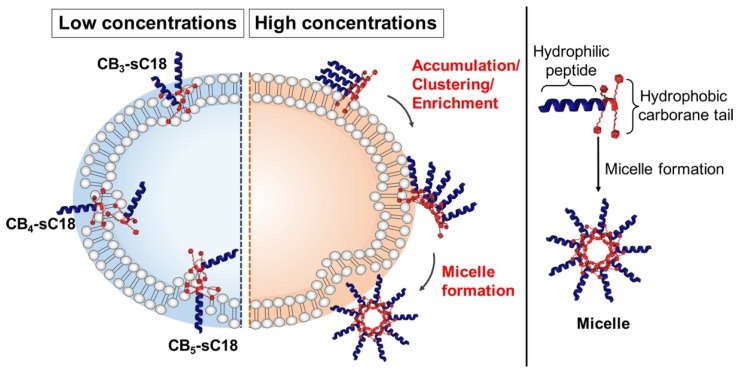
The hypothetical mechanism of interaction of CB-peptide conjugates with cellular membranes is demonstrated. Not shown is endocytosis, which we observed as one of the preferred internalization pathways (see Figure 3). Left: low concentrations of CB-CPP conjugates interact with the outer surface of the plasma membrane, leading to pore formation events and potential membrane distortion. Right: high concentrations of CB-peptide conjugates might result in potential self-assembly processes.

Our findings presented in this work prove that introducing CBs to cell-penetrating peptides modulates and enhances their membrane activity. We could demonstrate that the increased hydrophobicity caused by the attached CBs combined with the higher alpha-helical content significantly improved lipid phase interaction and thus, cellular uptake into HeLa cells for the CPP sC18. Beside their cellular uptake mediated by endocytosis, we assume that CB-CPPs intensively interact with lipid environments. More precisely, we hypothesize that CB-peptides, particularly those having more than two CBs included, tightly interact with lipid membranes in a way that they, in a first step, approach the membrane surface attracted by electrostatic interaction and facilitated by their amphipathic helix. Second and probably simultaneously, the CB tail is likely be inserted into the lipid core, tightly interacting with the fatty acid alkyl chains, and presumably sticking to it, inducing the following cell entry processes by formation of transient pores or inverted micelles. We cannot exclude that these interactions also lead to the recruitment of membrane components important for endocytotic uptake pathways. Since the CMC measured was above the concentration that was used in these experiments, we assume that CB-peptides contact the membrane surface in a singular state. However, we cannot completely exclude that self-assembled structures also play a role in membrane interaction, as displayed in Figure 5. In addition, we observed an intracellular vesicular distribution of the conjugates and therefore, we suppose that mainly endocytosis took place as the entry pathway at the concentrations applied. However, which pathway exactly is the favored one has to be elucidated in more detail in future studies.

Interestingly, our findings were transferable to the CPP pVEC, which was affected in the same manner as sC18 (Figure 1), concluding that CB coupling indeed alters the conformation of CPPs. Thus, the herein reported CB-CPP conjugates might present an interesting and valuable toolbox to study the first steps of CPP cellular uptake at the cellular membrane. More importantly, with the attachment of CB, one might further improve the overall performance of CPPs in terms of lipid–peptide interaction and internalization ability. Our results fit recent reports in which it was shown that peptides comprising high hydrophobic/amphipathic character induce membrane penetration and disrupting events when they insert deeply into the hydrophobic core of phospholipid membranes [38,39,40]. Additionally, we propose that in our case, the developed secondary structure played a crucial role for CB-CPP interplay and insertion within the lipid bilayer [41].

In line with these observations were the results obtained from our cellular studies. When increasing the applied conjugate concentrations, we observed increased cytotoxicity in HeLa cells as well as increased hemolytic activity in RBCs dependent on the number of CBs attached. However, all these effects were observed beyond a concentration threshold of 1 µM. Of note is that the new CB-conjugates were internalized highly efficiently and exhibited no cytotoxic effects when applied at lower concentrations of about 1 µM or below (Figure S33), which could make them interesting candidates for drug delivery applications.

Therefore, in preliminary studies, we investigated their performance as transport enhancers by simple co-incubation experiments. When incubating the anticancer drug daunorubicin with CB-sC18 conjugates, the activity of Dau was principally not diminished. Furthermore, CB_4_-sC18 showed slightly better activities compared to CB_1_-sC18, since it could slightly increase the cytotoxic activity of Dau (when co-incubated with 100 nM Dau). The fact that the preferred uptake of daunorubicin was not decreased in the presence of the peptides was possibly due to very fast uptake of the drug itself. Notably, we have seen a similar effect in previous studies, in which we used a cyclic peptidomimetic version of sC18 for enhancing the intracellular accumulation of daunorubicin. In this case, no improvement was detected when both substances were co-incubated and added to the cells [29]. On the other side, we investigated the ability of CB_1_-sC18 and CB_4_-sC18 in terms of nucleic acid delivery. Obviously, the transfection efficiency of the CB_1_-sC18/pDNA solution was only low, since only less cells showed a relatively weak signal after 72 h. Compared to this, we detected a clear strong red signal within cells that were transfected with the corresponding CB_4_-sC18/pDNA solution. Since the pDNA alone did not show any signal, we assume a successful delivery of the pDNA facilitated by CB_4_-sC18. In addition, we performed electro mobility shift assays (EMSA, see Figure S34) and observed that at the used pDNA/CB-CPP ratio, the pDNA was retarded. Thus, we conclude that a successful complex-like formation between both compounds is a prerequisite for successful transfection of the pDNA. Moreover, the intensity of the signal is comparable to the signal detected by the lipofectamine control (see Figure 4B and Figure S35), highlighting the high potential of CB-sC18 conjugates, particularly CB_4_-sC18, to act as transfection agent. 

In conclusion, we designed and synthesized a series of novel CB-CPP variants and analyzed how CB clusters influence the biophysical characteristics of CPPs. We found out that CBs affect secondary structure formation, probably leading to enhanced membrane activities. Therefore, the herein created new conjugates proved to be versatile tools for biological investigations, including, e.g., lipid–peptide interaction, as well as cargo delivery. In terms of further possible future studies, we highlight them as interesting candidates for application in BNCT therapy, where they might be ideal to transport high boron loads to the site of interest. In fact, as we have seen mainly endosomal accumulation of our CB-sC18 conjugates, the boron delivery to the perinuclear site might be significantly increased by making use of the endocytotic transport machinery.

## Figures and Tables

**Table 1:** Names, sequences, and analytical data of synthesized peptides. Peptides are *C*-terminally amidated. ***K***: lysine with CB attached at the ε-amino group. K: labeling position for CF.

Peptide	Sequence	MW_calc_. [Da]	MW_exp_. [Da]
sC18	^1^GLRKRLRKFRNKIKEK^16^	2069.55	2070.34
CB_1_-sC18	***K***-GLRKRLRKFRNKIKEK	2367.95	2368.74
CB_2_-sC18	***KK***-GLRKRLRKFRNKIKEK	2666.37	2666.97
CB_3_-sC18	***KKK***-GLRKRLRKFRNKIKEK	2965.79	2965.99
CB_4_-sC18	***KKKK***-GLRKRLRKFRNKIKEK	3264.21	3263.82
CB_5_-sC18	CB-***KKKK***-GLRKRLRKFRNKIKEK	3433.44	3434.29

**Table 2:** Percentage of α-helical content [31], and determined R-values of CB-sC18 and CB-pVEC conjugates calculated as the ratio between the molar ellipticity values at 222 and 208 nm [32].

Peptide	Phosphate Buffer ^1^	50% TFE ^1^	Phosphate Buffer ^2^	50% TFE ^2^	CMC [µM]
sC18	3.45	71.22	/	0.89	-
CB_1_-sC18	5.02	50.68	/	0.85	-
CB_2_-sC18	5.45	60.49	/	0.89	-
CB_3_-sC18	16.68	56.10	0.56	0.88	44.46 ± 1.14
CB_4_-sC18	21.68	56.30	0.73	0.98	43.45 ± 1.15
CB_5_-sC18	21.09	50.15	0.87	0.92	44.37 ± 1.16
pVEC	12.18	48.60	0.37	0.73	-
CB_1_-pVEC	17.72	44.28	0.57	0.82	-
CB_2_-pVEC	20.41	51.36	0.84	0.82	-
CB_3_-pVEC	16.98	65.76	0.99	0.82	-
CB_4_-pVEC	22.59	59.84	0.92	0.84	-
CB_5_-pVEC	22.96	45.48	0.79	0.79	-

^1^ % of alpha helix, ^2^ *R*-value.

## Supplementary Materials

The following are available online at https://www.mdpi.com/article/10.3390/pharmaceutics13122075/s1, Figure S1: UV-chromatogram and corresponding ESI-MS spectrum of sC18, Figure S2: UV-chromatogram and corresponding ESI-MS spectrum of CB_1_-sC18, Figure S3: UV-chromatogram and corresponding ESI-MS spectrum of CB_2_-sC18, Figure S4: UV-chromatogram and corresponding ESI-MS spectrum of CB_3_-sC18, Figure S5: UV-chromatogram and corresponding ESI-MS spectrum of CB_4_-sC18, Figure S6: UV-chromatogram and corresponding ESI-MS spectrum of CB_5_-sC18, Figure S7: UV-chromatogram and corresponding ESI-MS spectrum of CF-sC18 (pos. 12), Figure S8: UV-chromatogram and corresponding ESI-MS spectrum of CB_1_-CF-sC18, Figure S9: UV-chromatogram and corresponding ESI-MS spectrum of CB_2_-CF-sC18, Figure S10: UV-chromatogram and corresponding ESI-MS spectrum of CB_3_-CF-sC18, Figure S11: UV-chromatogram and corresponding ESI-MS spectrum of CB_4_-CF-sC18, Figure S12: UV-chromatogram and corresponding ESI-MS spectrum of CB_5_-CF-sC18, Figure S13: UV-chromatograms of all conjugates recorded using a linear gradient from 20–80% ACN in water (incl. 0.1% trifluoroacetic acid) within 15 min, Figure S14: Determination of critical micelle concentration (CMC) of *meta*-carboranyl-carboxylic acid, Figure S15: Determination of critical micelle concentration (CMC) of sC18, Figure S16: Determination of critical micelle concentration (CMC) of CB_1_-sC18, Figure S17: Determination of critical micelle concentration (CMC) of CB_2_-sC18, Figure S18: Determination of critical micelle concentration (CMC) of CB_3_-sC18, Figure S19: Determination of critical micelle concentration (CMC) of CB_5_-sC18, Figure S20: CD spectra of CB-sC18 conjugates (20 µM peptide concentration) in 10 mM phosphate buffer (pH 7.0) with the addition of 50% TFE, Figure S21: Helical wheel projections of CB-sC18 conjugates, Figure S22: (A) UV-chromatogram and (B) corresponding ESI-MS spectrum of pVEC after purification, Figure S23: (A) UV-chromatogram and (B) corresponding ESI-MS spectrum of CB_1_-pVEC after purification. Figure S24: (A) UV-chromatogram and (B) corresponding ESI-MS spectrum of CB_2_-pVEC after purification, Figure S25: (A) UV-chromatogram and (B) corresponding ESI-MS spectrum of CB_3_-pVEC after purification Figure S26: (A) UV-chromatogram and (B) corresponding ESI-MS spectrum of CB_4_-pVEC after purification, Figure S27: (A) UV-chromatogram and (B) corresponding ESI-MS spectrum of CB_5_-pVEC after purification, Figure S28: UV-chromatogram overlay of CB-pVEC conjugates, Figure S29: CD spectra of CB-pVEC conjugates (20 µM peptide concentration) in 10 mM phosphate buffer (pH 7.0) with the addition of 50% TFE, Figure S30: Hemolytic activity of carborane-peptide conjugates towards human red blood cells (RBCs), Figure S31: Cytotoxicity profile of *meta*-1-carborane-carboxylic acid towards HeLa cells, Figure S32: Cellular uptake of sC18 into HeLa cells via flow cytometry, Figure S33: Internalization of CB-sC18 conjugates after 30 min, Figure S34: Electrophoretic mobility shift assay (EMSA), Figure S35: Overlay of intensity profiles, Table S1: Names, sequences, analytical data and critical micelle concentration (CMC) of the CB-sC18 series, the CF-labeled CB-sC18 series and the CB-pVEC series.
