# Supplementary material for: Improving Membrane Activity and Cargo Delivery Efficacy of a Cell-Penetrating Peptide by Loading with Carboranes
Source: Pharmaceutics. 2021 Dec 3;13(12):2075. doi: 10.3390/pharmaceutics13122075 (PMC8709211; doi:10.3390/pharmaceutics13122075)
Supplement: Supplementary file 1 [file pharmaceutics-13-02075-s001.zip › pharmaceutics-1459726-supplementary.pdf]

pharmaceutics

Supplementary Materials: Improving Membrane Activity and Cargo Delivery Efficacy of a Cell-Penetrating Peptide by Loading with Carboranes

Tamara Lützenburg, Nele Burdina, Matthias S. Scholz and Ines Neundorf

Table S1. Names, sequences, analytical data and critical micelle concentration (CMC) of the CB-sC18 series, the CF-labeled CB-sC18 series and the CB-pVEC series. Peptides are C-terminally amidated. *K*: lysine with CB attached at the ε-amino group. K: labeling position for CF.

Peptide	Sequence	MW$_{calc.}$ [Da]	MW$_{exp.}$ [Da]	Purity [%]	CMC [μM]
sC18	^{1}GLRKRLRKFRNKIKEK^{16}	2069.55	2070.34	>99	-
CB$_1$-sC18	*K*-GLRKRLRKFRNKIKEK	2367.95	2368.74	>99	n.d.
CB$_2$-sC18	*KK*-GLRKRLRKFRNKIKEK	2666.37	2666.97	>95	n.d.
CB$_3$-sC18	*KKK*-GLRKRLRKFRNKIKEK	2965.79	2965.99	>97	44.46 ± 1.14
CB$_4$-sC18	*KKKK*-GLRKRLRKFRNKIKEK	3264.21	3263.82	>98	43.45 ± 1.15
CB$_5$-sC18	CB-*KKKK*-GLRKRLRKFRNKIKEK	3433.44	3434.29	>96	44.37 ± 1.16
CF-sC18	^{1}GLRKRLRKFRNKIKEK^{16}	2427.87	2429.06	>99	n.d
CB$_1$-CF-sC18	*K*-GLRKRLRKFRNKIKEK	2726.27	2727.47	>99	n.d
CB$_2$-CF-sC18	*KK*-GLRKRLRKFRNKIKEK	3024.68	3025.79	>95	n.d
CB$_3$-CF-sC18	*KKK*-GLRKRLRKFRNKIKEK	3323.08	3324.37	>96	n.d
CB$_4$-CF-sC18	*KKKK*-GLRKRLRKFRNKIKEK	3621.48	3623.16	>98	n.d
CB$_5$-CF-sC18	CB-*KKKK*-GLRKRLRKFRNKIKEK	3791.71	3793.26	>97	n.d
pVEC	^{1}LLIILRRRIRKQAHAHSK^{18}	2209.70	2208.92	>97	n.d
CB$_1$-pVEC	*K*-LLIILRRRIRKQAHAHSK	2507.11	2506.63	>99	n.d
CB$_2$-pVEC	*KK*-LLIILRRRIRKQAHAHSK	2805.51	2805.78	>99	n.d
CB$_3$-pVEC	*KKK*-LLIILRRRIRKQAHAHSK	3103.91	3105.91	>98	n.d
CB$_4$-pVEC	*KKKK*-LLIILRRRIRKQAHAHSK	3402.31	3402.37	>99	n.d
CB$_5$-pVEC	CB-*KKKK*-LLIILRRRIRKQAHAHSK	3572.54	3572.44	>97	n.d

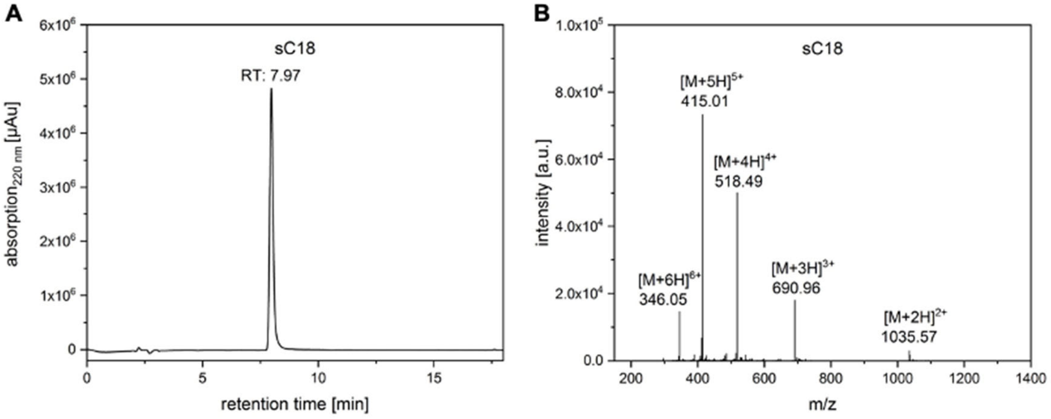

Figure S1. (**A**) UV-chromatogram and (**B**) corresponding ESI-MS spectrum of sC18 after purification. UV-chromatogram was recorded using a linear gradient from 10–60% ACN in water (incl. 0.1% TFA) over 15 min.

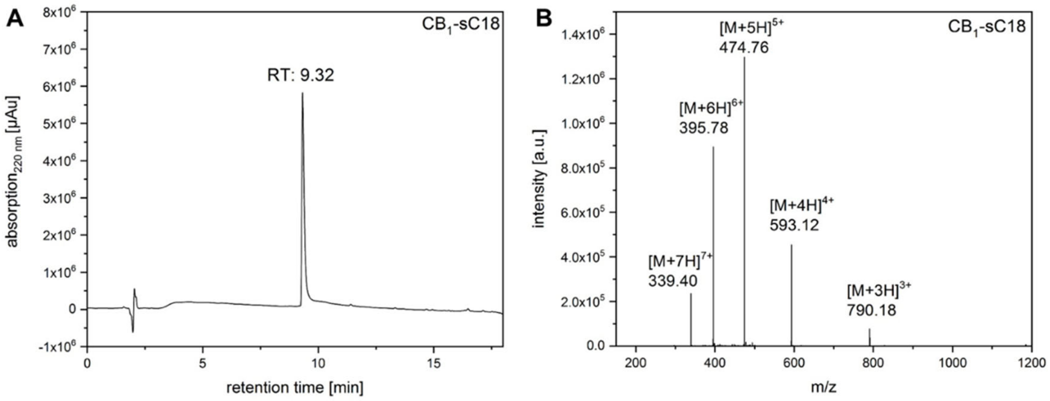

Figure S2. (**A**) UV-chromatogram and (**B**) corresponding ESI-MS spectrum of CB_1-sC18 after purification. UV-chromatogram was recorded using a linear gradient from 10-60% ACN in water (incl. 0.1% TFA) over 15 min.

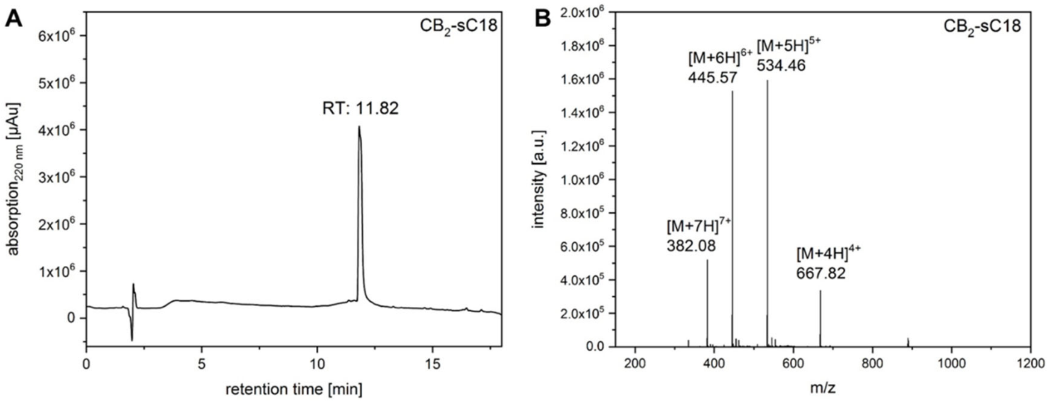

Figure S3. (**A**) UV-chromatogram and (**B**) corresponding ESI-MS spectrum of CB_2-sC18 after purification. UV-chromatogram was recorded using a linear gradient from 10–60% ACN in water (incl. 0.1% TFA) over 15 min.

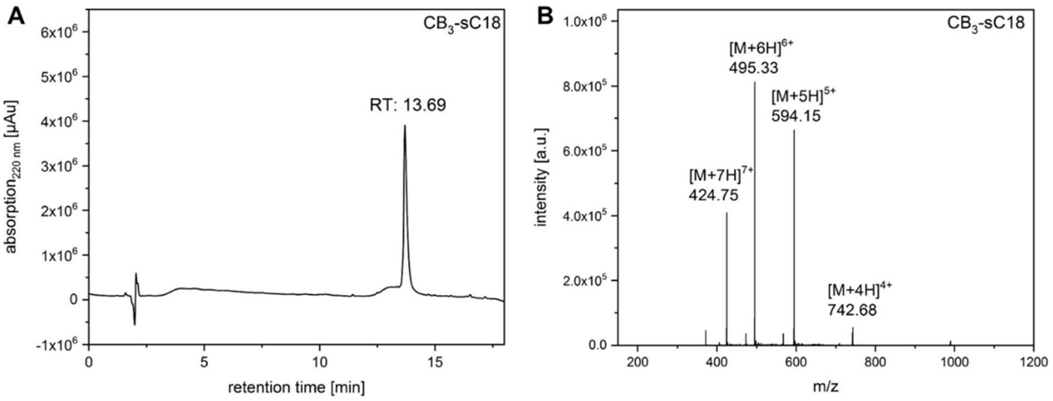

Figure S4. (**A**) UV-chromatogram and (**B**) corresponding ESI-MS spectrum of CB_3-sC18 after purification. UV-chromatogram was recorded using a linear gradient from 10–60% ACN in water (incl. 0.1% TFA) over 15 min.

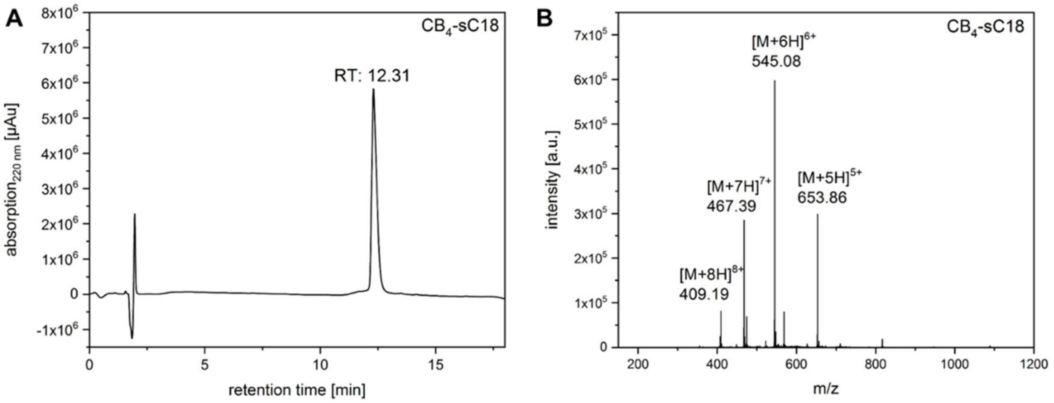

Figure S5. (**A**) UV-chromatogram and (**B**) corresponding ESI-MS spectrum of CB_4-sC18 after purification. UV-chromatogram was recorded using a linear gradient from 20–70% ACN in water (incl. 0.1% TFA) over 15 min.

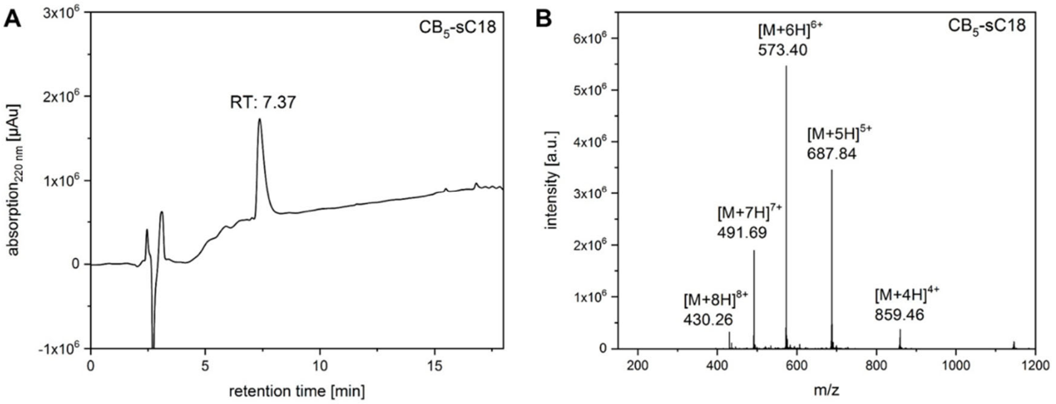

Figure S6. (**A**) UV-chromatogram and (**B**) corresponding ESI-MS spectrum of CB_5-sC18 after purification. UV-chromatogram was recorded using a linear gradient from 30–90% ACN in water (incl. 0.1% TFA) over 15 min.

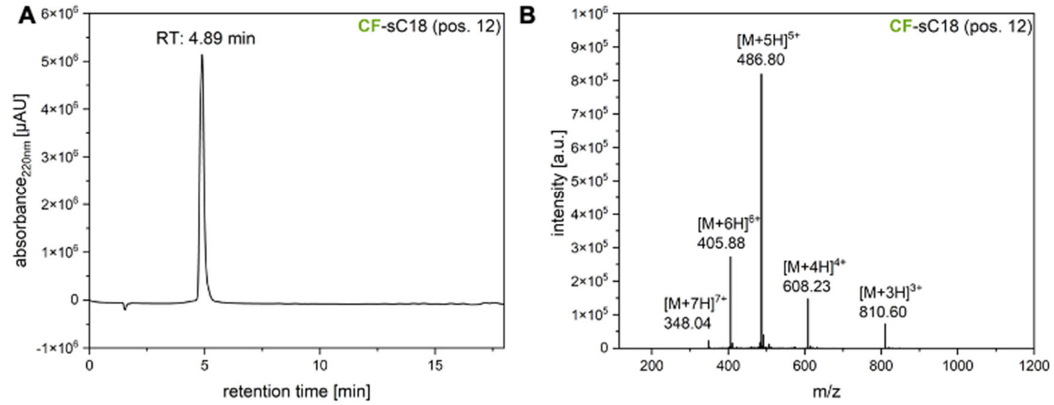

Figure S7. (**A**) UV-chromatogram and (**B**) corresponding ESI-MS spectrum of CF-sC18 (pos. 12) after purification. UV-chromatogram was recorded using a linear gradient from 20–80% ACN in water (incl. 0.1% TFA) over 15 min.

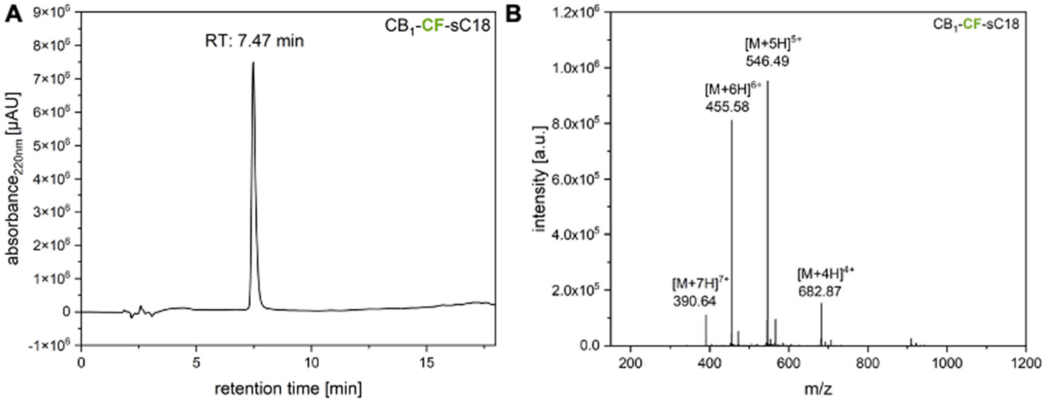

Figure S8. (**A**) UV-chromatogram and (**B**) corresponding ESI-MS spectrum of CB_1-CF-sC18 after purification. UV-chromatogram was recorded using a linear gradient from 20–80% ACN in water (incl. 0.1% TFA) over 15 min.

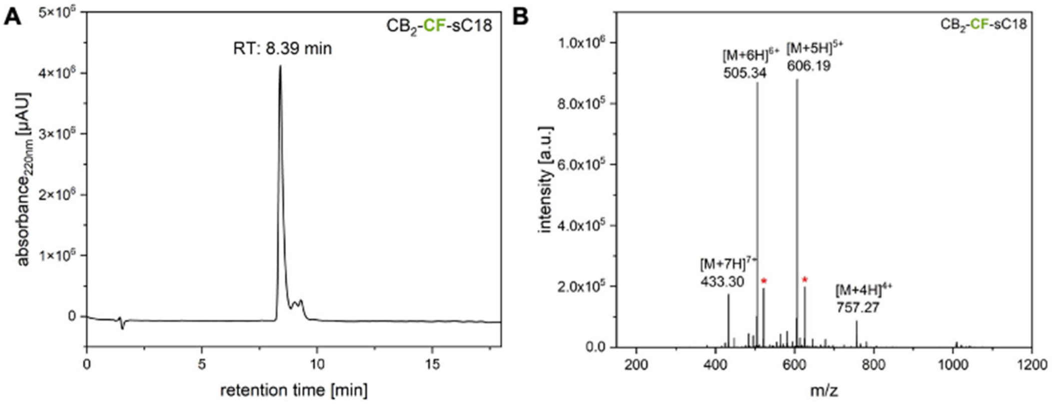

Figure S9. (**A**) UV-chromatogram and (**B**) corresponding ESI-MS spectrum of CB_2-CF-sC18 after purification. UV-chromatogram was recorded using a linear gradient from 20–80% ACN in water (incl. 0.1% TFA) over 15 min. Red stars indicate TFA-adducts, which appear in the UV-chromatogram as a shoulder.

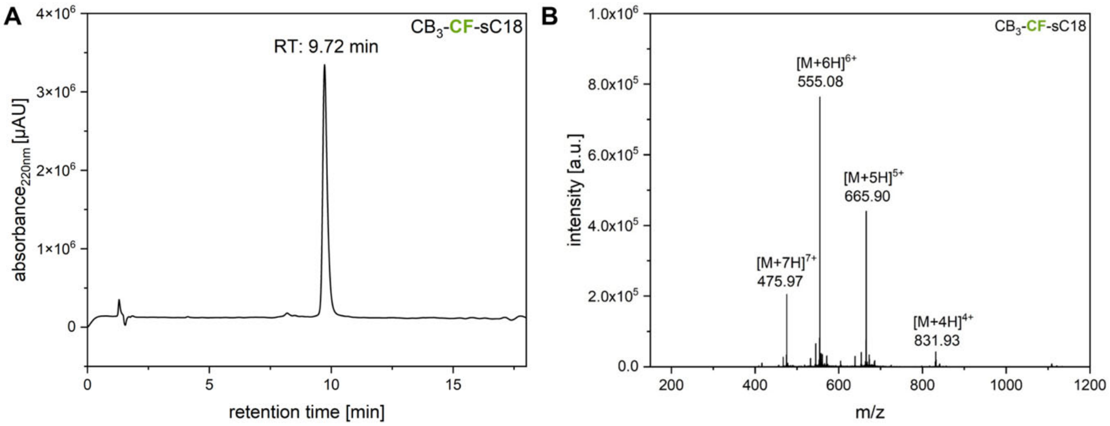

Figure S10. (**A**) UV-chromatogram and (**B**) corresponding ESI-MS spectrum of CB_3-CF-sC18 after purification. UV-chromatogram was recorded using a linear gradient from 20–80% ACN in water (incl. 0.1% TFA) over 15 min.

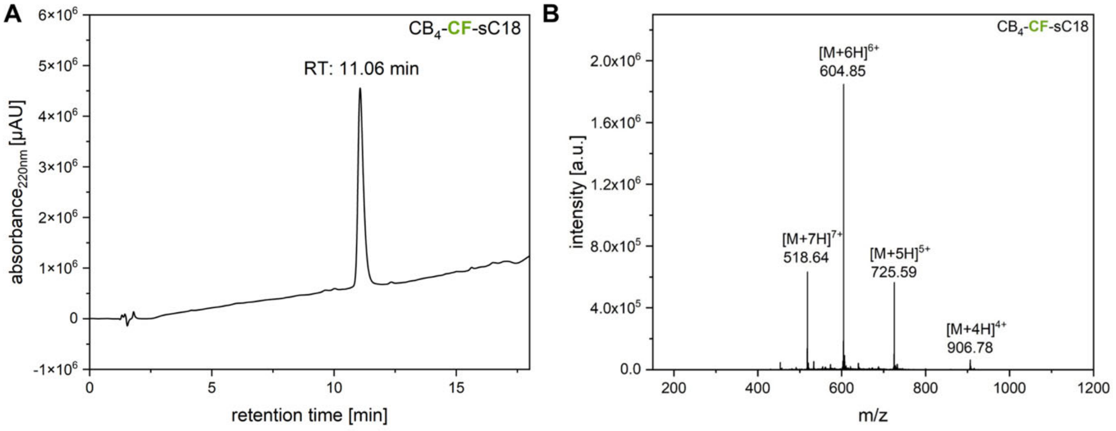

Figure S11. (**A**) UV-chromatogram and (**B**) corresponding ESI-MS spectrum of CB_4-CF-sC18 after purification. UV-chromatogram was recorded using a linear gradient from 20–80% ACN in water (incl. 0.1% TFA) over 15 min.

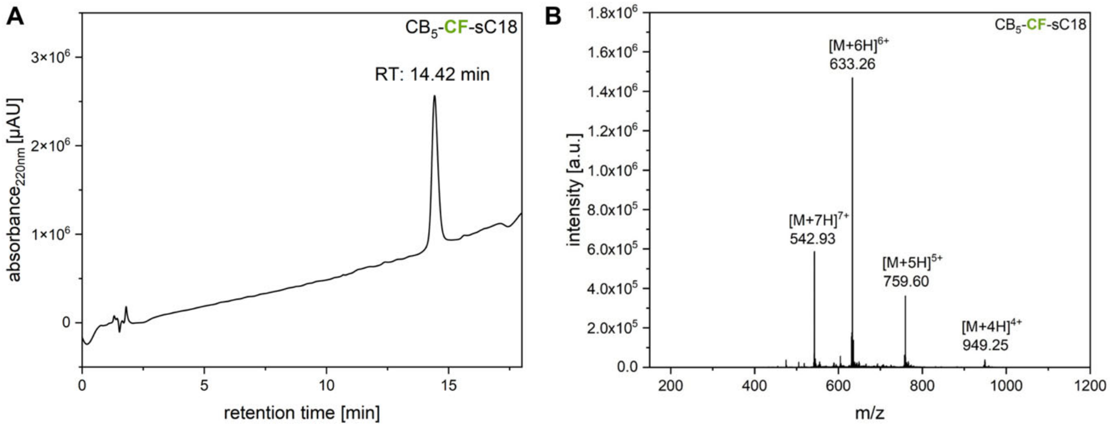

Figure S12. (**A**) UV-chromatogram and (**B**) corresponding ESI-MS spectrum of CB_5-CF-sC18 after purification. UV-chromatogram was recorded using a linear gradient from 20–80% ACN in water (incl. 0.1% TFA) over 15 min.

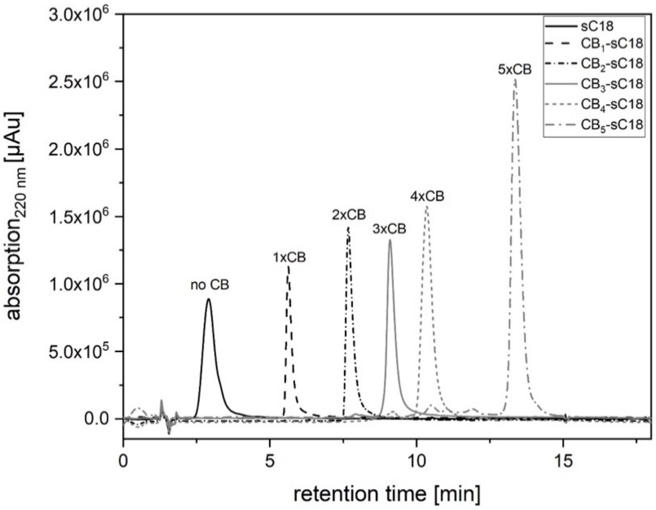

Figure S13. UV-chromatograms of all conjugates recorded using a linear gradient from 20–80% ACN in water (incl. 0.1% TFA) within 15 min. Retention times: sC18: 2.91 min; CB_1-sC18: 5.63 min; CB_2-sC18: 7.67 min; CB_3-sC18: 9.10 min; CB_4-sC18: 10.35 min; CB_5-sC18: 13.36 min.

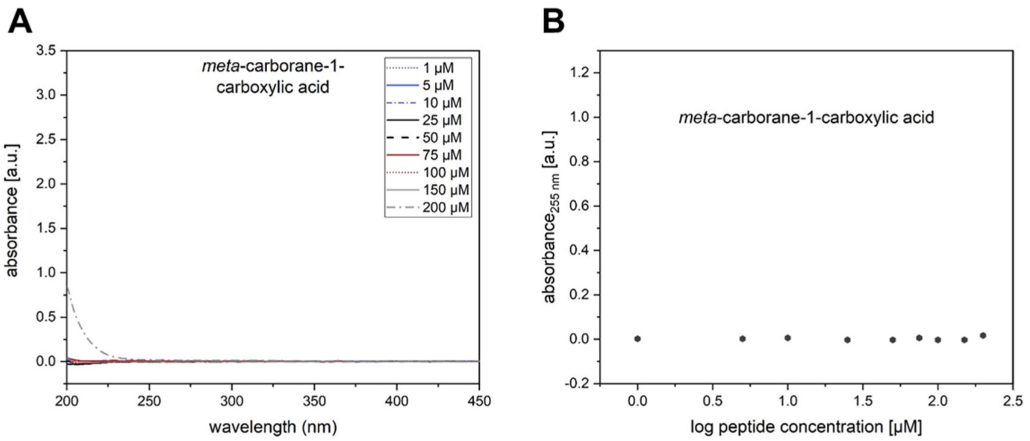

Figure S14. Determination of critical micelle concentration (CMC) of *meta*-carboranyl-carboxylic acid. (**A**) UV-VIS spectrum from 200–450 nm of *meta*-carboranyl-carboxylic acid at various concentrations (1–200 μM). (**B**) Absorbance at 255 nm plotted against the logarithm of peptide concentration.

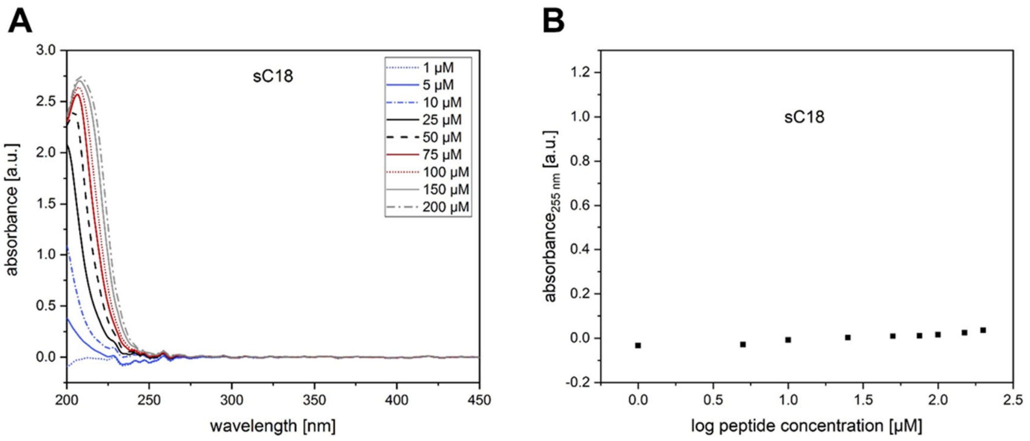

Figure S15. Determination of critical micelle concentration (CMC) of sC18. (**A**) UV-VIS spectrum from 200–450 nm of sC18 at various concentrations (1–200 μM). (**B**) Absorbance at 255 nm plotted against the logarithm of peptide concentration.

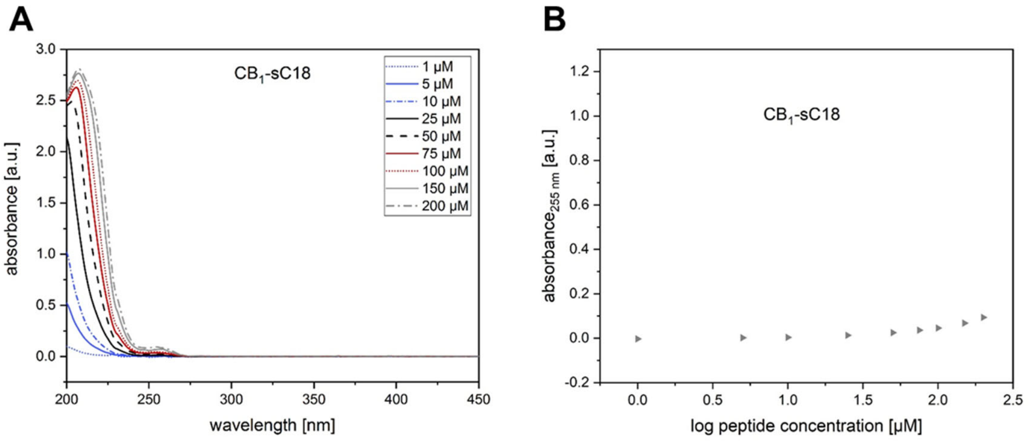

Figure S16. Determination of critical micelle concentration (CMC) of CB_1-sC18. (**A**) UV-VIS spectrum from 200–450 nm of CB_1-sC18 at various concentrations (1–200 μM). (**B**) Absorbance at 255 nm plotted against the logarithm of peptide concentration.

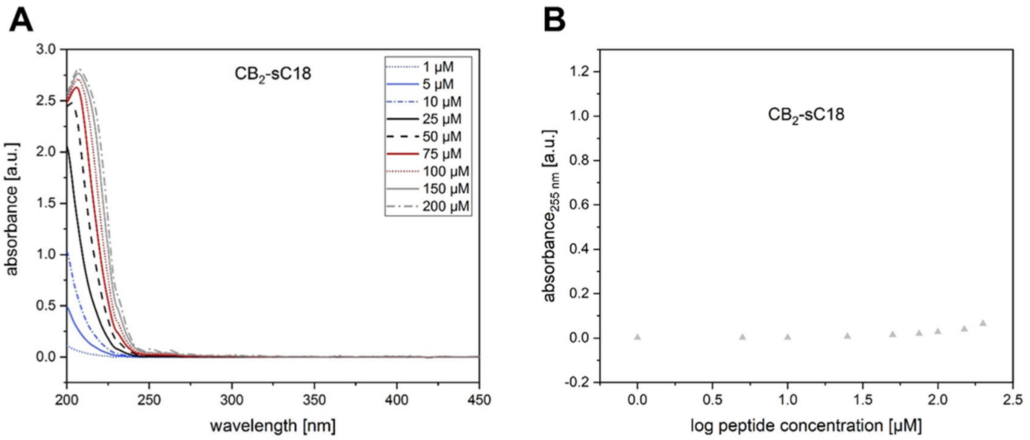

Figure S17. Determination of critical micelle concentration (CMC) of CB_2-sC18. (**A**) UV-VIS spectrum from 200–450 nm of CB_2-sC18 at various concentrations (1–200 µM). (**B**) Absorbance at 255 nm plotted against the logarithm of peptide concentration.

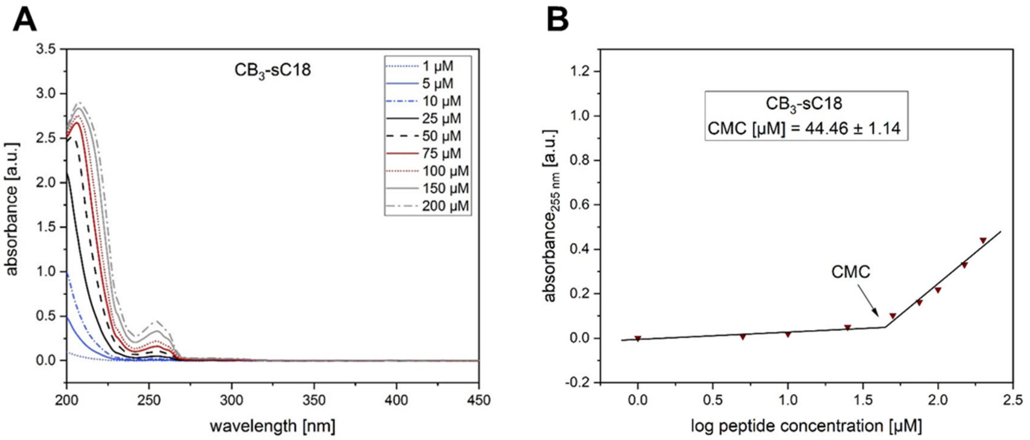

Figure S18. Determination of critical micelle concentration (CMC) of CB_3-sC18. (**A**) UV-VIS spectrum from 200–450 nm of CB_3-sC18 at various concentrations (1–200 µM). (**B**) Absorbance at 255 nm plotted against the logarithm of peptide concentration. The intersection point indicates the CMC.

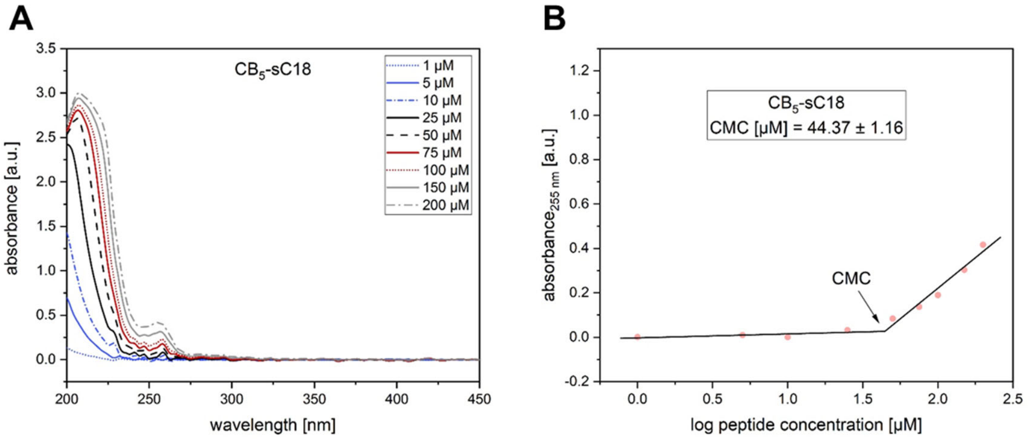

Figure S19. Determination of critical micelle concentration (CMC) of CB_5-sC18. (**A**) UV-VIS spectrum from 200–450 nm of CB_5-sC18 at various concentrations (1–200 µM). (**B**) Absorbance at 255 nm plotted against the logarithm of peptide concentration. The intersection point indicates the CMC.

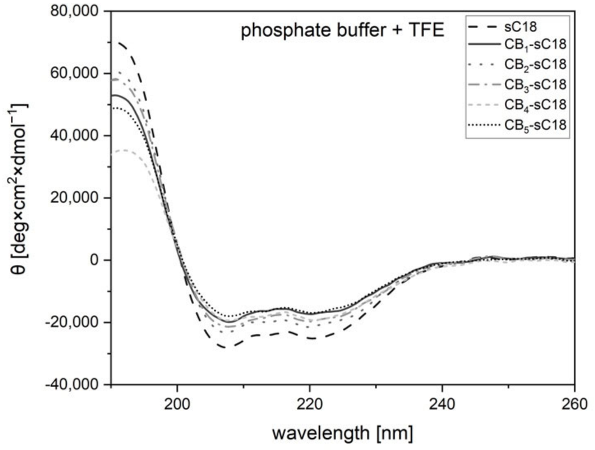

Figure S20. CD spectra of CB-sC18 conjugates (20 μM peptide concentration) in 10 mM phosphate buffer (pH 7.0) with the addition of 50% TFE.

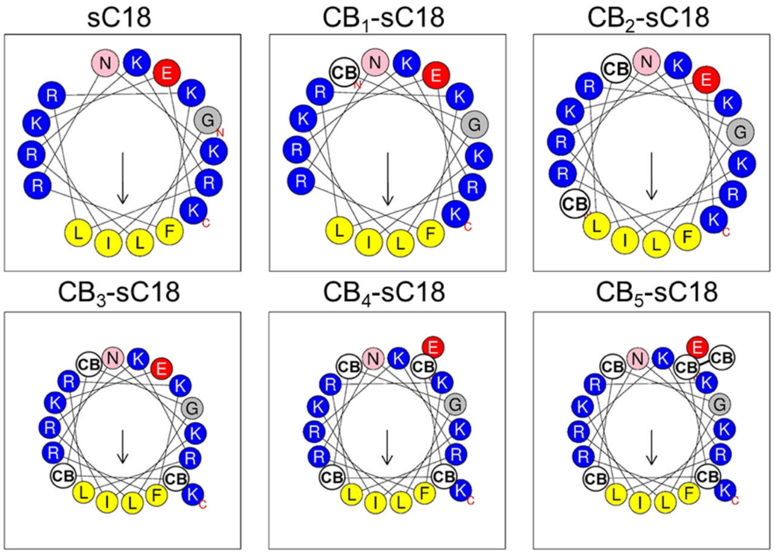

Figure S21. Helical wheel projections of CB-sC18 conjugates. Blue-filled circles represent positively charged residues, red-filled circles negatively charged residues. Non-polar residues are highlighted in yellow, uncharged in grey. CB represents *meta*-carboranyl-carboxylic acid.

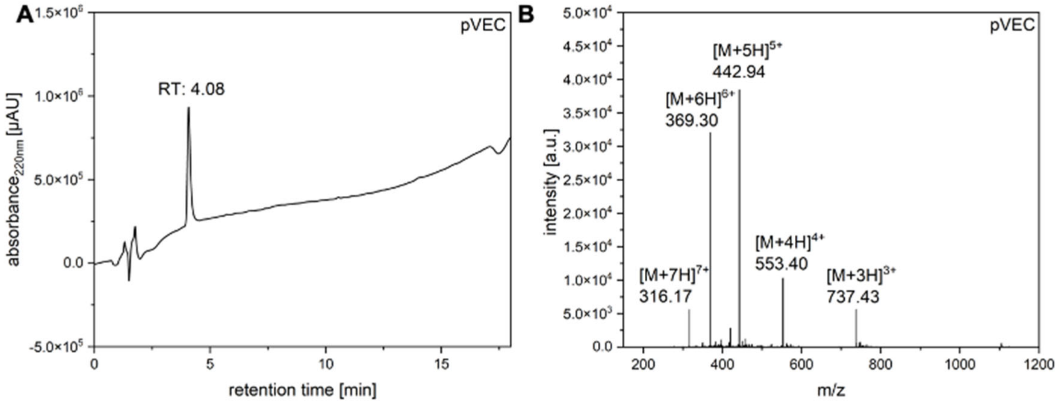

Figure S22. (**A**) UV-chromatogram and (**B**) corresponding ESI-MS spectrum of pVEC after purification. UV-chromatogram was recorded using a linear gradient from 20–80% ACN in water (incl. 0.1% TFA) over 15 min.

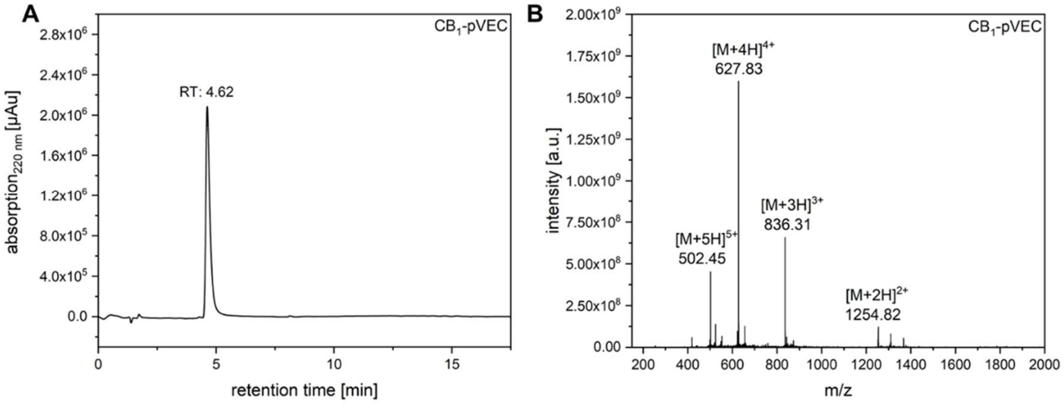

Figure S23. (**A**) UV-chromatogram and (**B**) corresponding ESI-MS spectrum of CB_1-pVEC after purification. UV-chromatogram was recorded using a linear gradient from 20–80% ACN in water (incl. 0.1% TFA) over 15 min.

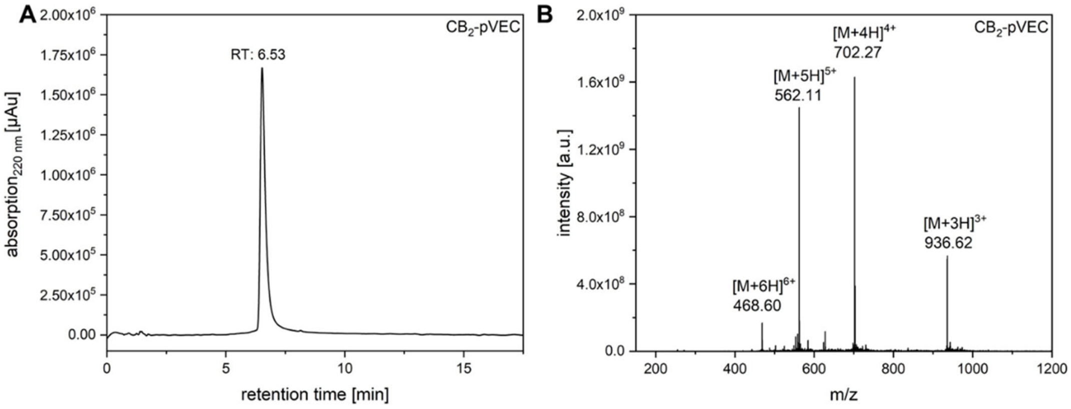

Figure S24. (**A**) UV-chromatogram and (**B**) corresponding ESI-MS spectrum of CB_2-pVEC after purification. UV-chromatogram was recorded using a linear gradient from 20–80% ACN in water (incl. 0.1% TFA) over 15 min.

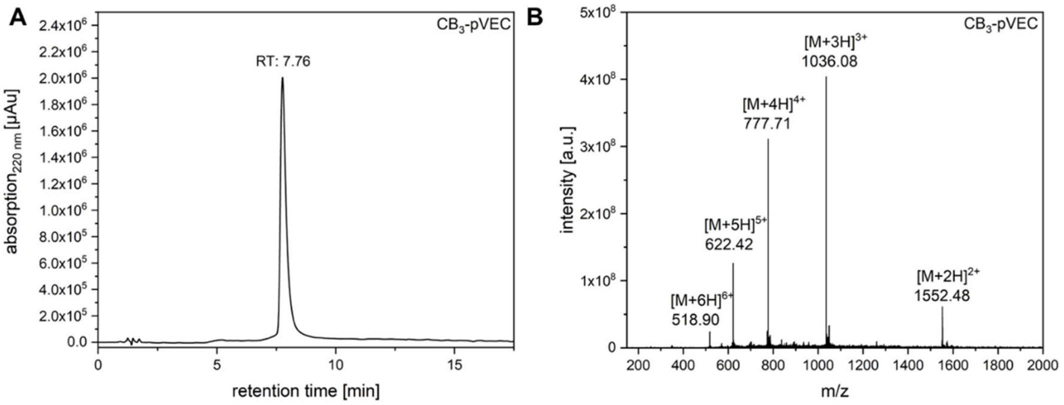

Figure S25. (**A**) UV-chromatogram and (**B**) corresponding ESI-MS spectrum of CB_3-pVEC after purification. UV-chromatogram was recorded using a linear gradient from 20–80% ACN in water (incl. 0.1% TFA) over 15 min.

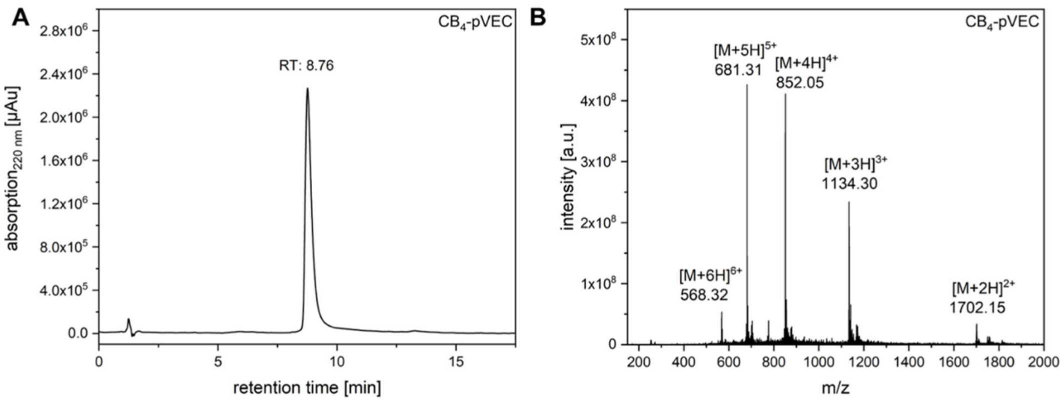

Figure S26. (**A**) UV-chromatogram and (**B**) corresponding ESI-MS spectrum of CB_4-pVEC after purification. UV-chromatogram was recorded using a linear gradient from 20–80% ACN in water (incl. 0.1% TFA) over 15 min.

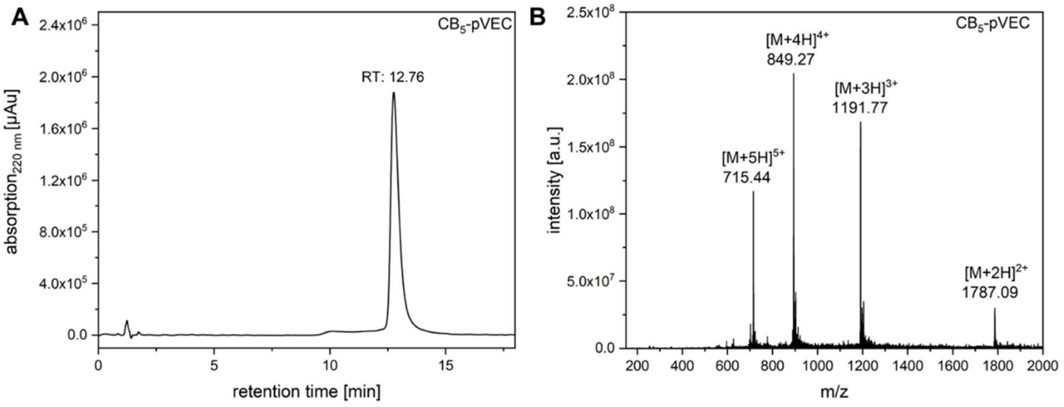

Figure S27. (**A**) UV-chromatogram and (**B**) corresponding ESI-MS spectrum of CB_5-pVEC after purification. UV-chromatogram was recorded using a linear gradient from 20–80% ACN in water (incl. 0.1% TFA) over 15 min.

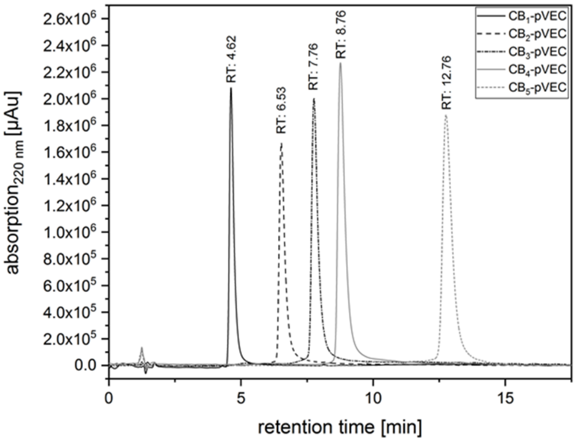

Figure S28. UV-chromatogram overlay of CB-pVEC conjugates. UV-chromatogram was recorded using a linear gradient from 20–80% ACN in water (incl. 0.1% TFA) over 15 min.

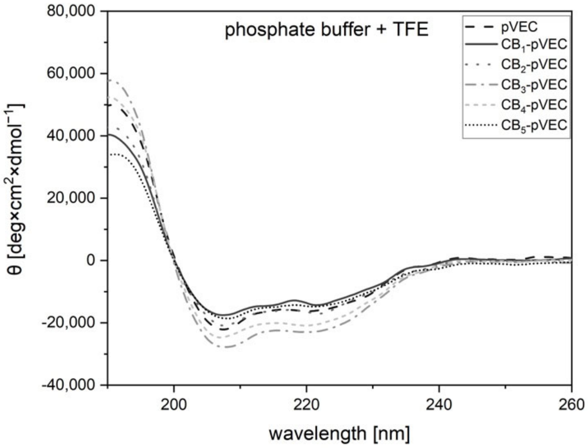

Figure S29. CD spectra of CB-pVEC conjugates (20 µM peptide concentration) in 10 mM phosphate buffer (pH 7.0) with the addition of 50% TFE.

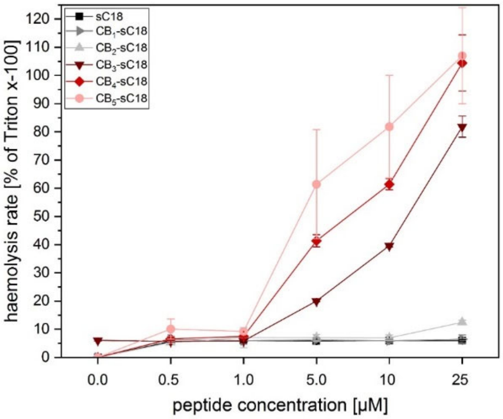

Figure S30. Hemolytic activity of carborane-peptide conjugates towards human red blood cells (RBCs). RBCs were treated with different peptide concentrations (0.5–25 µM) for 1 h. RBCs treated with Triton X-100 served as control. Data were normalized to control. Experiments were conducted in triplicate with $n = 2$. Error bars represent standard deviation.

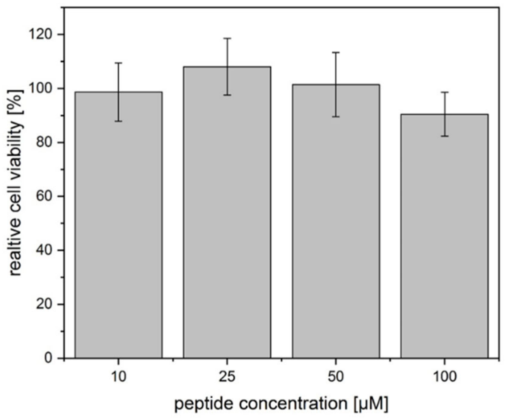

Figure S31. Cytotoxicity profile of *meta*-1-carborane-carboxylic acid towards HeLa cells. Compound was incubated for 24 h at different concentrations (10–100 µM) with HeLa cells. Data were normalized to untreated cells (100% viability). Cells treated with 70% ethanol served as positive control. Experiments were performed in triplicate with $n = 2$. Error bars represent standard deviation.

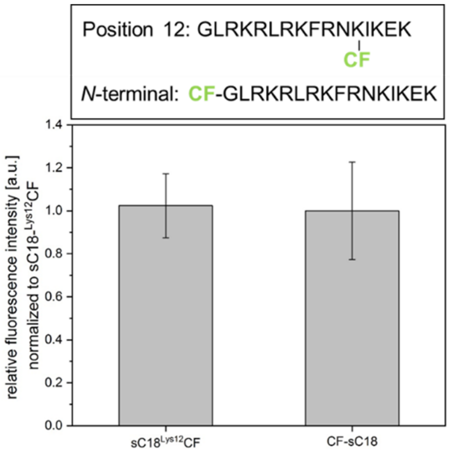

Figure S32. Cellular uptake of sC18 into HeLa cells via flow cytometry. The fluorophore 5(6)-Carboxyfluorscein was either attached at the *N*-terminus or position 12 of sC18. Peptides were incubated for 30 min. Experiments were conducted in triplicate with $n = 3$. Error bars represent standard deviation.

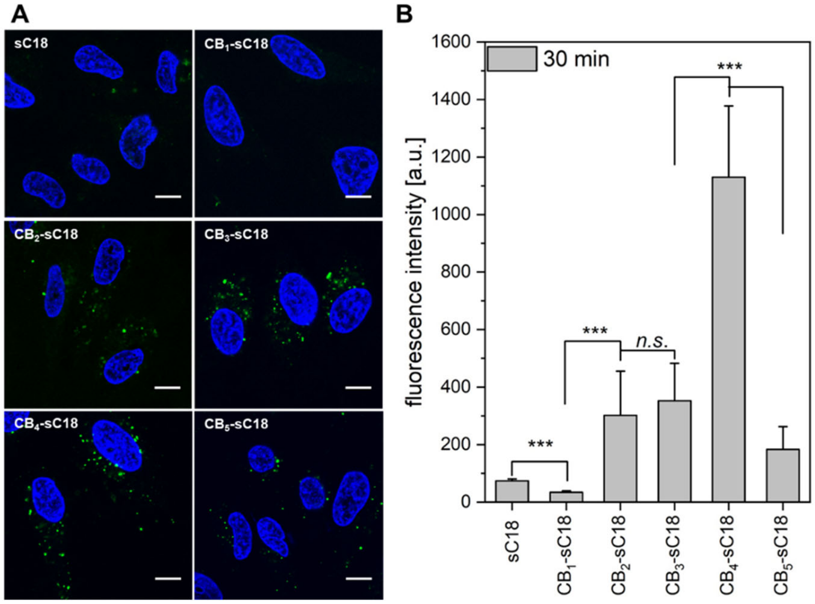

Figure S33. Internalization of CB-sC18 conjugates after 30 min. (**A**) Confocal laser scanning microscopic analysis of 1 µM CF-labeled peptide conjugates after 30 min incubation time; green: 5(6)-Carboxyfluorescein-labeled peptide conjugates; blue: Hoechst 33342 nuclear stain; scale bar: 10 µm. (**B**) Corresponding flow cytometry analysis of 1 µM CF-labeled peptide conjugates for 30 min. Experiments were conducted in triplicate with $n = 3$. Error bars represent standard deviation. Significances were determined by Student's t-test (*** $p < 0.0005$). CB_4-sC18 is significant (*** $p < 0.0005$) to all conjugates.

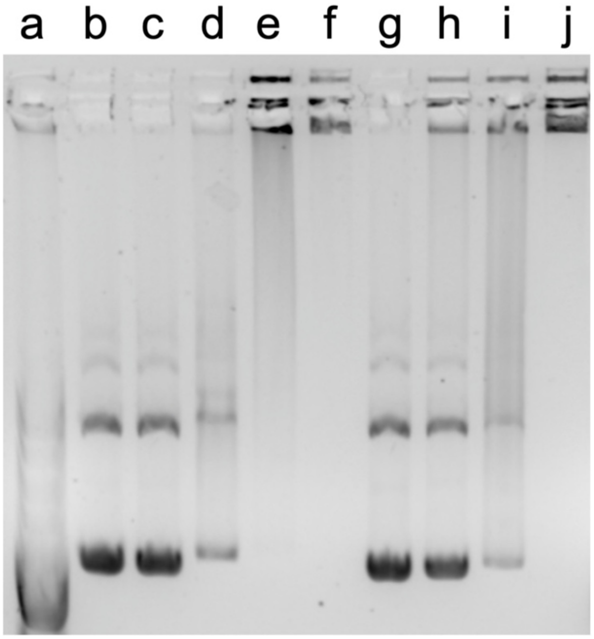

Figure S34. Electrophoretic mobility shift assay (EMSA). mCherry plasmid and peptides CB_1-sC18/CB_4-sC18 were incubated at different mass ratios for 1h at 37 °C and analyzed via agarose gel electrophorese. The agarose gel was run at 100 V for 1.5 h. For the mCherry plasmid a defined amount of 250 ng (1 = 250 ng) was used. Loading of lanes: a: 1kb DNA ladder; b: mCherry plasmid, c: 1:0.25 CB_1-sC18; d: 1:0.5 CB_1-sC18; e: 1:1 CB_1-sC18; f: 1:3 CB_1-sC18, g: 1:0.25 CB_4-sC18; h: 1:0.5 CB_4-sC18; i: 1:1 CB_4-sC18; j: 1:3 CB_4-sC18.

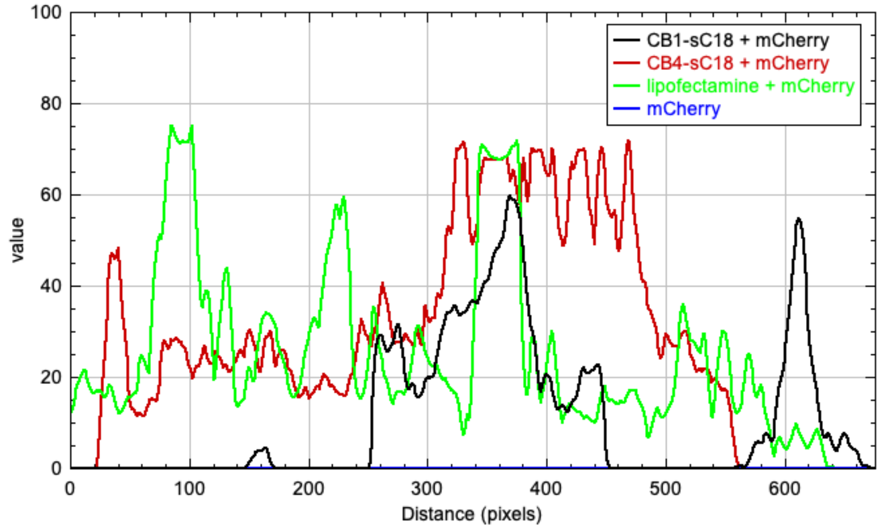

Figure S35. Overlay of intensity profiles. Shown are the intensity profiles as overlay of CB_1-sC18 + mCherry (black), CB_4-sC18 + mCherry (red), lipofectamine + mCherry (green) and mCherry plasmid alone (blue).
